# The Three-Day Treatment of Drug and Alcohol Habitues with Hyoscine

**Published:** 1910-01

**Authors:** H. V. Riewel

**Affiliations:** Cleveland, Ohio


### Abstracts and Selections.

### The Three-Day Treatment of Drug and Alcohol Habitues With Hyoscine.

BY H. V. RIEWEL, M. D., CLEVELAND, OHIO.

The name hyoscine was first applied by Ladenburg in 1880 to an alkaloid of hyoscyamus possessing nerve depressant, mydriatic and hypnotic properties. Individual patients show a varying degree of susceptibility or idiosyncrasy and tolerance for the drug as may be seen in the following: P. S. Root reports a case of poisoning from a single hypodermic dose of hyoscine hydrobromate of 1-300 grain; W. A. Carey, three cases from a dose of 1-100 grain; L. W. Morton, one case from 1-75 grain hyoscine, five minutes after injection.

On the other hand marked tolerance for the drug is shown by the following instances: During active treatment of one of H. G. Wagner's cases, after physiological effects had been obtained by the usual method, one-tenth grain of hyoscine was given by mistake without any ill effects. W. H. H. Githens reports a case of accidental dose by mouth of four-fifths grain hyoscine without ill effects.

*A Resume of the Literature on the Treatment of the Drug Habit by Hyoscine Hydrobromate.*—The first to have successfully and openly used it was M. K. Lott,* who reported 25 cases of the morphine habit thus treated in 1901, and again in 1902 he reported 34 cases. J. M. Buchanan reported 12 cases, treated by hyoscine, at the proceedings of the American Psychological Association in 1903, and L. Abramson, 20 cases of the drug habit treated in this way. He wrote me, that before giving up the method he used it in 100 cases with no deaths. Sixty of the one hundred remained permanently free from the use of drugs. Forty per cent relapsed.

R. E. Behring reported six cases of the morphine habit and four

*Texas Medical Journal, November, 1902.

alcoholic addicts successfully treated. He considers it as specific in the morphine habit as antitoxin is for diphtheria. A. W. Richardson one case of the morphine habit treated by the hyoscine method; J. M. Catchings, accurately and in detail, 15 cases of the opium habit treated with hyoscine; H. G. Wagner, 5 cases of the drug habit and 7 cases of the liquor habit. The latter two authors give the most reliable and accurate course to pursue. I prefer the method described by Wagner. It seems to me the less dangerous of the two, because of the small amount of hyoscine necessary in a given case, due to its combination with atropine and strychnine, which I believe are somewhat supportive and therefore safer. Catching's method of a demonstration is practically the same, except that he uses hyoscine alone and uncombined with other drugs.

The most remarkable series of cases treated by this method, but not yet reported, I have learned of in a letter from C. C. Stockard. He has treated 800 cases of the various drug habits during the past ten years by the hyoscine method, but of late uses it only in patients who suffer too much pain from the gradual withdrawal of the morphine which he finds to be about one in four, or 25 per cent of all cases. He mentions two deaths in this series, one from pneumonia, the second caused by perforating appendicitis. No deaths occurred in cases treated by other observers mentioned.

Oscar Jennings, of Le Vesinet, France, in a letter writes that his first and only attempt at treatment of a case of opium habit with hyoscine was in 1888. He was unsuccessful, consequently he considered it too dangerous. After hearing of its success in America, he was prompted to use hyoscine upon himself. He took it by the method described below until he obtained the physiological effects. This forced him to conclude that the drug was not as dangerous as it first seemed.

*Method Employed with Report of Cases.*—In the drug cases the method employed has been the same as that described by Wagner, i. e., hypodermic medication of from forty-eight to seventy-two hours' duration. The alcoholic cases were more favorable subjects, treated at their homes with competent attendants. The drugs were given by mouth during the first eight days, just enough to keep the throat dry and pupils dilated, as for example 1-100 grain to 1-50 grain hyoscine—1-500 grain atropine and 1-60 to 1-30 grain strychnine, every two to four hours. During the ninth and tenth days the hypodermic was used, pushing the treatment to the stage of mild delirium. In the alcoholic cases the delirium was of but two days' duration. The patients were males without abnormal physical findings.

Ten cases of the liquor habit were treated by this method. Four have not relapsed to date. The longest period of total abstinence after treatment is three years. Treatment for this case ended December 7, 1905. The other three patients have abstained, one for six months, the other two for nine months each. Of the six relapsed cases the shortest period before relapse was three months. Not one of these returned to the liquor habit because of the craving for drink, but simply to take one drink socially then let it alone. Thus the desire was created and they relapsed within four to ten months after treatment.

*Ten Morphine Cases Treated by the Hyoscine Method.*—For the privilege of reporting four of these cases I am indebted to Dr. Wagner, they were treated by him at the Cleveland City Hospital. As an example of the average course, a detailed report of one case will be given, including bedside chart. This case is interesting from a surgical standpoint as well.

Mr. J., high school principal, referred to me by Dr. F. C. Herrick, 34 years old, had been addicted to the use of morphine for eight years, beginning in 1899 when it was administered to relieve the pain of gall-stone disease. Family history was negative. Physical examination was negative except moderate anemia and slight tenderness on deep pressure over the gall-bladder. The urine was acid, specific gravity 1024, contained no sugar, no bile, nor albumin. The morphine taken had consisted of four grains per dose by hypodermic four times daily, making sixteen grains every twenty-four hours. An initial dose of calomel grains three, followed by Rochelle salts one ounce, was given. Special nurse day and night during the first week. Regular hospital vigilance during the second week. Active treatment began June 2, 1908, at 4 p. m., when a single hypodermic was given, consisting of hyoscine hydrobromate, grain 1-200; atropine, grain 1-600, and strychnine, grain 1-200, in distilled water. This was repeated every hour and a half for eight doses. Then one-half this dose was given for the six succeeding periods of one and a half hours each. This was followed by twelve full doses at one and a half hour intervals ending the active treatment with two half doses. The last hypodermic of hyoscine was given June 5th at 2:30 p. m. Altogether during the active treatment, which lasted sixty-nine hours, one-fourth grain hyoscine, one-fourth grain strychnine and one-twenty-fourth grain atropine were administered. A copy of the bedside record is appended giving in detail the treatment and management.

After the first week patients as a rule eat heartily and sleep normally; the appetite becomes ravenous usually about the tenth day. Those who complain of insomnia may for one or two nights, after first four days' active treatment, be relieved by any suitable hypnotic: trional, grains 20, or chloral and bromides, of each grains 15, for one or two doses at bedtime. There is no craving for the drug nor pain nor suffering from its withdrawal at any time during or after treatment. Occasionally one finds a patient who sleeps most of the time during the three days of active treatment. These should not be pushed to the stage of mild delirium described on the bedside record. This delirium referred to should be carefully controlled since too large doses at this time will create a wild, almost unmanageable, delirium with attempts to crawl up the wall, etc. This, however, can be controlled at any time when the patient becomes unmanageable, by giving one-fourth grain of morphine, which will not in any way interfere with the results of treatment.

In mild delirium, the delusions and illusions are altogether quite pleasant, leaving no bad effects. In this patient, who had been a soldier in the Spanish War, while looking intently at the figures on the wall paper, they suddenly became transformed into troops of marching soldiers. He would look at the chandelier watching turners' and acrobatic performances.

A quite common illusion is mistaking a white counterpane for black broadcloth which the patient is buying for his wife and children for clothing. Sometimes the sheet is torn into shreds in making endless yards of cloth for purchase. A smoker will reach into space for his pipe which, when he is about to grasp it, suddenly disappears.

In looking out of the window, one of the men saw a tree which suddenly expanded into a beautiful park. He intended to walk out of the second story window into the park, with lakes and benches scattered here and there. He was easily dissuaded upon being asked to sit down on this bench (chair in the room). He did so still looking out into the tree when he whispered to me to watch a pair of lovers on yonder bench. He said at first they were sitting far apart, but now he was moving closer and closer with his arm about her waist. Here his delusions were stopped by suggesting that he was looking at a tree. "Only a tree?" "Yes, yes." "Why, I saw them a minute ago, now they have vanished." The park and lovers disappeared as suddenly as they appeared. Others will walk about picking up rings, etc., from empty space and hiding them under the pillow.

This patient's delirium was very carefully adjusted, so that he was kept in bed most of the time, part of the time asleep, some time answering his wife or talking to his daughter. Then again the nurse's white apron would bring about a conversation with the butcher.

At this stage too much hyoscine will make them wildly delirious. Walking about, sometimes jumping upon the window sill or table. This can be controlled by one-fourth grain morphine, or if the pulse is good they can easily be led about the room and put back to bed. They are quite amenable to suggestions at this time and if watched they will without the dose of morphine become quieter and more tractable within an hour or two. The effects of the drug disappearing entirely within six to twenty-four hours after treatment is discontinued. During this stage of active treatment, sneezing and vomiting occur very commonly.

*Case of Opium Poisoning in Baby Three Months Old.*—July 19, 1905, I was called at 5 a. m. to see a boy three months old. Examination showed pin point pupils with no reaction to light, lids half open; respiration four to six a minute and irregular, sometimes completely arrested for from fifteen to thirty seconds; hands and lower extremities cold, skin and mucous membranes cyanotic. The evening before, the child's mother borrowed some soothing mixture from an accommodating neighbor. The boy seemed in severe pain, so she gave him a teaspoonful every little while, how often she could not recall, until the child was sound asleep. He slept all night. Parents tried unsuccessfully for an hour by slapping, shaking, hot and cold water baths, etc., to rouse him, but the stupor became deeper. Shortly after my arrival a hypodermic injection, containing strychnine grain 1-1000, hyoscine grain 1-1000 and atropine grain 1-2000, was given. Two more doses were given fifteen minutes apart. Twenty minutes after the third dose pupils became larger with flushed cheeks. Thirty minutes later breathing was twenty times a minute. At this time teaspoonfuls of water were swallowed. The same medication was then continued by mouth for three days, always watching for physiological effects. The necessity for continuing treatment for three days was shown when the child would fall into a deep sleep if the interval between doses was too long, especially during the first forty-eight hours. This seemed to show that the effect of hyoscine disappears quite rapidly, but that morphine is eliminated rather slowly. The text-books, I believe, coincide with this statement. The boy is at present living and in good health; now three and one-half years old.

*Conclusions.*—1. The hyoscine treatment will eliminate the desire of drug and alcohol habitues for these drugs, thus eliminating the element which prevents the patients abstaining by force of will power.

2. That having lost the desire they do very well without intoxicants or the drugs as shown by the increase in appetite, gain in flesh and their general improvement.

3. The question of relapse lies entirely in the sincerity and environment of the patient.

4. The favorable alcoholic addicts are those who earnestly desire to discontinue the use of intoxicants and are willing to change their mode of living and environment; but who can not until relieved of the craving for liquor.

5. Relapse in both drug and liquor cases is not due to a desire nor suffering after the treatment, but to their curiosity to test the necessity of total abstinence, or to the temptations of social life.

6. That a single dose of the drug or drink of liquor, even after one year of total abstinence, is very apt to start the craving resulting in a condition which is no better than before treatment.

7. This method may prove a valuable treatment for apparently hopeless cases of opium poisoning. Interesting experiments along this line might be carried out.

8. The one contraindication for this treatment is the presence of Bright's disease.

9. That no case should be treated unless put to bed and watched by competent nurses day and night during the first week.

Bibliography—Omitted on account of lack of space.—*Reprinted from Monthly Cyclopedia and Medical Bulletin,* October, 1909.

